# Risk factors for bleeding complications in patients undergoing extracorporeal cardiopulmonary resuscitation following out-of-hospital cardiac arrest: a secondary analysis of the SAVE-J II study

**DOI:** 10.1186/s13613-024-01253-x

**Published:** 2024-01-28

**Authors:** Shutaro Isokawa, Toru Hifumi, Keita Hirano, Yu Watanabe, Katsuhiro Horie, Kijong Shin, Kasumi Shirasaki, Masahiro Goto, Akihiko Inoue, Tetsuya Sakamoto, Yasuhiro Kuroda, Shiori Tomita, Norio Otani, The SAVE-J II study group

**Affiliations:** 1 Department of Emergency and Critical Care Medicine, St. Luke’s International Hospital, 9-1 Akashicho, Chuo-Ku, Tokyo, 104-8560 Japan; 2 Department of Human Health Sciences, Graduate School of Medicine, Kyoto University, Kyoto, Japan; 3 Department of Emergency and Critical Care Medicine, Hyogo Emergency Medical Center, Kobe, Japan; 4 Department of Emergency Medicine, Teikyo University School of Medicine, Tokyo, Japan; 5 Department of Emergency Medicine, Kagawa University School of Medicine, Kagawa, Japan; 6 Tama Family Clinic, Kanagawa, Japan

**Keywords:** Bleeding, Complication, Extracorporeal cardiopulmonary resuscitation, Platelet count, Out-of-hospital cardiac arrest, Risk factor

## Abstract

**Background:**

Bleeding is the most common complication in out-of-hospital cardiac arrest (OHCA) patients receiving extracorporeal cardiopulmonary resuscitation (ECPR). No studies comprehensively described the incidence rate, timing of onset, risk factors, and treatment of bleeding complications in OHCA patients receiving ECPR in a multicenter setting with a large database. This study aimed to analyze the risk factors of bleeding during the first day of admission and to comprehensively describe details of bleeding during hospitalization in patients with OHCA receiving ECPR in the SAVE-J II study database.

**Methods:**

This study was a secondary analysis of the SAVE-J II study, which is a multicenter retrospective registry study from 36 participating institutions in Japan in 2013–2018. Adult OHCA patients who received ECPR were included. The primary outcome was the risk factor of bleeding complications during the first day of admission. The secondary outcomes were the details of bleeding complications and clinical outcomes.

**Results:**

A total of 1,632 patients were included. Among these, 361 patients (22.1%) had bleeding complications during hospital stay, which most commonly occurred in cannulation sites (14.3%), followed by bleeding in the retroperitoneum (2.8%), gastrointestinal tract (2.2%), upper airway (1.2%), and mediastinum (1.1%). These bleeding complications developed within two days of admission, and 21.9% of patients required interventional radiology (IVR) or/and surgical interventions for hemostasis. The survival rate at discharge of the bleeding group was 27.4%, and the rate of favorable neurological outcome at discharge was 14.1%. Multivariable logistic regression analysis showed that the platelet count (< 10 × 10^4^/μL vs > 10 × 10^4^/μL) was significantly associated with bleeding complications during the first day of admission (adjusted odds ratio [OR]: 1.865 [1.252–2.777], p = 0.002).

**Conclusions:**

In a large ECPR registry database in Japan, up to 22.1% of patients experienced bleeding complications requiring blood transfusion, IVR, or surgical intervention for hemostasis. The initial platelet count was a significant risk factor of early bleeding complications. It is necessary to lower the occurrence of bleeding complications from ECPR, and this study provided an additional standard value for future studies to improve its safety.

**Supplementary Information:**

The online version contains supplementary material available at 10.1186/s13613-024-01253-x.

## Background

Although the efficacy of extracorporeal cardiopulmonary resuscitation (ECPR) in patients with out-of-hospital cardiac arrest (OHCA) was recently examined [1–3], its safety should also be considered. Despite the great development of ECPR, bleeding is still its most common complication in OHCA patients [4–12]. Belohlavek et al. reported that there were major bleeding occurred in 36 patients (31%) treated with an invasive strategy including ECPR, and 10 patients (15%) received conventional cardiopulmonary resuscitation, and fatal bleeding occurred in 4 patients (11%) and non (0%), respectively [1]. In the Chinese ECMO registry that includes ECPR, patients with any bleeding complications had a significantly higher in-hospital mortality rate than those without [12].

Several studies discussed the clinical characteristics and risk factors of bleeding complications in a small number of OHCA patients who underwent ECPR [4–10, 13]. Parameters related to bleeding complications, such as anticoagulation therapy, blood transfusion status, and laboratory data, were also not discussed [12]. Thus, no studies comprehensively described the incidence rate, timing of onset, risk factors, and treatment of bleeding complications in OHCA patients who received ECPR in a multicenter setting with a large database.

Therefore, this study aimed to examine the risk factors and details of bleeding complications in patients with OHCA who received ECPR using a dataset from 36 institutions in Japan.

## Methods

### SAVE-J II study

The SAVE-J II study was a multicenter retrospective registry study conducted in Japan, with 36 participating institutions [14]. The study aimed to investigate the outcomes of OHCA patients who received ECPR [14]. The study was registered at the University Hospital Medical Information Network Clinical Trials Registry and the Japanese Clinical Trial Registry (registration number: UMIN000036490) and approved by the institutional review board of Kagawa University (approval number: 2018–110) and each participating institution, including St. Luke’s International Hospital (approval number: 18-R188).

### Study design and participants

This study performed a secondary analysis of the SAVE-J II study [14], which included patients aged 18 years or older who presented with OHCA, were admitted to the emergency department between January 1, 2013, and December 31, 2018, and received ECPR (resuscitation for cardiac arrest with venoarterial extracorporeal membrane oxygenation [VA-ECMO]). The exclusion criteria were as follows: patients who received VA-ECMO after intensive care unit (ICU) admission, patients who were withdrawn after cannulation due to the return of spontaneous circulation (ROSC), patients with external causes of cardiac arrest, such as acute aortic dissection/aortic aneurysm, hypothermia, primary cerebral disorders, infection, drug intoxication, trauma, suffocation, and drowning, patients who achieved ROSC at hospital arrival and ECMO initiation, patients who transferred from other hospitals, patients with unknown outcomes and cannula insertion failure, and patients without records of bleeding complications. This secondary analysis was conducted with the approval of the institutional review board of St. Luke's International Hospital (approval number: 21-R135), and the requirement for informed consent was waived. All procedures were performed in accordance with the ethical standards of the review board of St. Luke's International Hospital on human experimentation and with the Helsinki Declaration of 1975.

### Data collection

The following information of ECPR patients in SAVE-J II was collected: age, sex, comorbidities, antiplatelet/anticoagulant medications taken regularly prior to hospital admission, location of cardiac arrest, occurrence of witnessed cardiac arrest and bystander cardiopulmonary resuscitation (CPR), initial cardiac rhythm at the scene and prior to ECMO initiation, time course, laboratory data obtained upon arrival, place of ECMO cannulation, volume of blood transfusion within 48 h after ICU admission, coronary angiography (CAG), percutaneous catheter intervention (PCI), interventional radiology (IVR), surgical intervention for hemostasis, etiology of cardiac arrest, length of ICU stay, length of hospital stay, in-hospital mortality, neurological outcomes, and occurrence, and site of bleeding complications.

### Definitions

The time from call ambulance to arrival was defined as time from the call to emergency medical services until hospital arrival. The estimated low flow time was defined as the time from cardiac arrest to the establishment of ECMO if the location of cardiac arrest was in an ambulance and the time from calling an ambulance to the establishment of ECMO if cardiac arrest happened in other location. Cardiac arrest was either due to cardiac causes like acute coronary syndrome, arrhythmia, myopathy, and myocarditis, or non-cardiac causes like pulmonary embolism.

### Bleeding complications

First, bleeding complications during hospitalization, not limited to the first 48 h after admission in this registry, were assessed. Bleeding complications were defined as cases requiring blood transfusion, IVR, or surgical hemostasis, except for cases of cerebral hemorrhage confirmed by CT scan. Bleeding sites were cannula sites, retroperitoneum, puncture sites (excluding ECMO cannulation site), the brain, upper airway, chest (including mediastinal bleeding, hemothorax, pulmonary hemorrhage, etc.), abdomen (including gastrointestinal tract bleeding, liver, spleen, and abdominal cavity), and other sites. Blood transfusions apparently required for the consumption through ECMO device were not defined (included) as bleeding complications.

### Prehospital resuscitation efforts by emergency medical service personnel and the details of ECPR implementation in Japan

A previous study showed the details of the emergency medical service (EMS) system in Japan [15]. Prehospital termination of resuscitation by EMS personnel is not allowed unless patients have evident signs of death (i.e., rigor mortis and postmortem lividity). Thus, the EMS personnel in Japan must perform resuscitation and transport patients with OHCA to the hospital with continuous resuscitation efforts until the achievement of ROSC or upon hospital arrival. As for the details of ECPR initiation, a previous study reported differences in ECPR practice in patients with OHCA from the emergency room (ER) to the ICU [16]. In two-thirds of institutions, emergency physicians perform cannulation. The catheterization room was the most common location of cannulation (48.6%), followed by the ER (31.4%). Vascular access was obtained via percutaneous through ultrasonography (47.2%), followed by percutaneous without ultrasonography (44.4%) [16].

### Outcome measures

The primary outcome was the risk factor of bleeding complications during the first day of admission. The secondary outcomes were the details of bleeding complications, such as incidence rate, timing of onset, treatment requiring blood transfusion, IVR, or/and surgical interventions for hemostasis in patients undergoing ECPR. Moreover, clinical outcomes, such as survival at hospital discharge, neurological outcomes, and length of ICU stay were evaluated.

### Statistical analysis

Continuous variables are presented as medians with interquartile ranges (IQRs), while categorical variables are presented as numbers and percentages (%). The hazards of bleeding were calculated as proportions of patients with bleeding complications among those who were alive initially. Mann–Whitney-Wilcoxon test and chi-squared test were used to compare continuous and categorical variables between the two groups, respectively. The mixed-effects logistic regression model was used to evaluate the influence of multiple predictors on the binary outcome. This model includes both observed covariates (fixed effects) and inter-group variability (random effects). In particular, the random effect was incorporated to account for variations in center effect across different facilities. The multivariate analysis was adjusted for age, comorbidities, medications, estimated low flow time, hemoglobin, platelets, fibrinogen, ECMO cannulation place, and each institution, which were considered as clinically relevant and previously reported as risk factors for bleeding complications [10, 17–21]. To assess the robustness of our risk factor study, additional analyses were conducted with composite outcome including bleeding complication and death during the first day of admission, and with competing risk analysis using Fine-Gray model accounting for early mortality during the first week of admission. The odds ratios (ORs) with 95% confidence intervals were calculated. *P*-values of < 0.05 were considered statistically significant. All statistical analyses were conducted using JMP Pro 17.0.0 (SAS, the USA) and R software version 4.1.1 (R Foundation for Statistical Computing, Vienna, Austria). Missing data were not replaced or estimated.

## Results

**Fig. 1:**
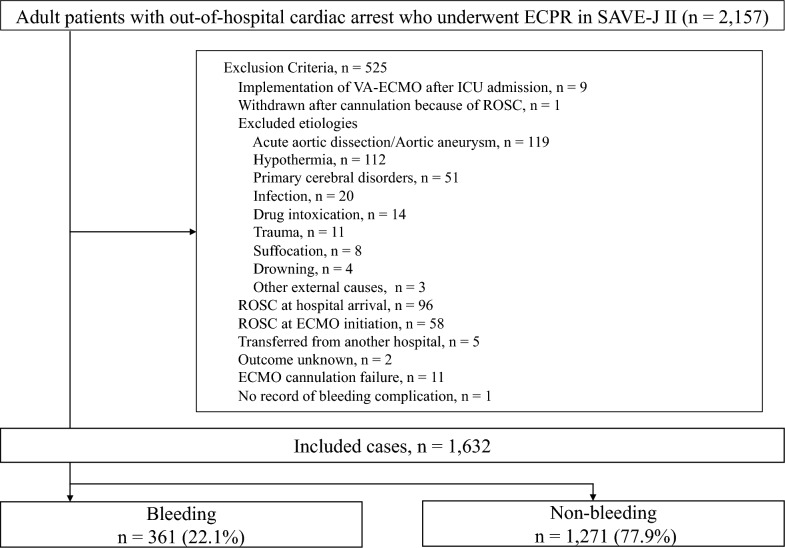
Patient selection flow chart. ECPR, extracorporeal cardiopulmonary resuscitation; VA-ECMO, venoarterial extracorporeal membrane oxygenation; ICU, intensive care unit; ROSC, return of spontaneous circulation

A patient selection flow chart is shown in Fig. 1. Of the 2,157 adult patients with OHCA who received ECPR in SAVE-J II, 1,632 patients were included in this study, of which 361 patients (22.1%) developed bleeding complications (bleeding group) during hospitalization, while 1,271 (77.9%) did not (non-bleeding group).

### Characteristics of eligible patients

The study population consisted of 1,632 patients, with a median age of 60 years (IQR, 49–68), and 84.7% were men. Among them, 16.1% were regularly taking antiplatelet or anticoagulant medications, while 2.2% were taking both regularly. The median time from call ambulance to arrival was 32 min (IQR, 26–39), and the estimated low flow time was 55 min (IQR, 45–66). Cannulation was performed in an ER, catheter laboratory, and other places in 64.0%, 35.8%, and 0.2%, respectively. On arrival, the median hemoglobin level was 12.9 g/dL (IQR, 11.0–14.6), platelet count was 14.7 × 10^4^/µL (IQR, 10.9–19.0), fibrinogen level was 215 mg/dL (IQR, 164–277), and D-dimer level was 14.3 μg/mL (IQR, 5.7–31.4). The etiology of cardiac arrest was cardiogenic (86.4%), non-cardiogenic (6.5%), and unknown (7.1%). The median length of ICU stay was 3 (IQR: 1–10) days. The rates of survival discharge and favorable neurological outcome upon hospital discharge were 27.4% and 14.1%, respectively. In a univariate analysis of the bleeding and non-bleeding groups during hospitalization, significant differences were observed in terms of age, time from call ambulance to arrival, platelet count upon arrival, rhythm before ECMO initiation, ECMO cannulation place, red blood cell administration, blood product transfusion volume from arrival to 48 h after ICU admission, CAG, PCI, and etiology of cardiac arrest. The non-bleeding group had a significantly longer length of ICU and hospital stay than the bleeding group (median: 7 vs. 2 days, p < 0.001; 9 vs. 3 days, p < 0.001, respectively). The non-bleeding group had a higher survival at discharge than the bleeding group (34.9% vs 25.3%, p < 0.001). No significant difference was observed in terms of the favorable neurological outcomes at discharge from the hospital (13.9% vs 14.2%, p = 0.881) (Table 1).

**Table 1:** Characteristics and comparisons between patients with and without bleeding complications during hospitalization^a^

	All n = 1632	Bleeding^b^ n = 361	Non-bleeding n = 1,271	P-value
Age, years	60 (49–68)	62 (50–70)	59 (49–68)	**0.010**
Sex, male	1382 (84.7)	306 (84.8)	1076 (84.7)	0.960
Comorbidity				
Hypertension	501 (32.3)	114 (32.8)	387 (32.1)	0.829
Diabetes mellitus	332 (21.4)	79 (22.7)	253 (21.0)	0.499
Dyslipidemia	196 (12.6)	53 (15.2)	143 (11.9)	0.097
Cardiovascular disease	417 (26.9)	97 (27.9)	320 (26.6)	0.631
Cerebrovascular disease	100 (6.4)	29 (8.3)	71 (5.9)	0.103
Chronic renal failure	82 (5.3)	18 (5.2)	64 (5.3)	0.916
Medication				0.122
No antiplatelet and anticoagulant	1153 (81.7)	267 (81.0)	886 (82.0)	
Antiplatelet or anticoagulant	227 (16.1)	51 (15.5)	176 (16.3)	
Antiplatelet and anticoagulant	31 (2.2)	12 (3.6)	19 (1.8)	
Location of cardiac arrest				0.917
Home	649 (39.9)	137 (38.0)	512 (40.4)	
Workplace	179 (11.0)	41 (11.4)	138 (10.9)	
Public place	287 (17.6)	64 (17.8)	223 (17.6)	
Street	230 (14.1)	49 (13.6)	181 (14.3)	
Witnessed by emergency medical service	182 (11.2)	45 (12.5)	137 (10.8)	
Others	100 (6.1)	24 (6.7)	76 (6.0)	
Witnessed cardiac arrest	1278 (78.7)	283 (79.1)	995 (78.7)	0.872
Bystander CPR	925 (57.6)	208 (58.6)	717 (57.3)	0.656
Initial cardiac rhythm				0.496
Shockable	1125 (69.6)	251 (69.7)	874 (69.6)	
PEA	366 (22.6)	86 (23.9)	280 (22.3)	
Asystole	125 (7.7)	23 (6.4)	102 (8.1)	
Time from call ambulance to arrival (min) ^c^	32 (26–39)	30 (24–37)	32 (26–39)	**0.005**
Estimated low flow time (min) ^d^	55 (45–66)	54 (43–66)	55 (46–66)	0.186
Laboratory dates				
Hemoglobin (g/dL)	12.9 (11.0–14.6)	13.0 (10.9–14.7)	12.9 (11.1–14.6)	0.863
Platelet (× 10^4^/μL)	14.7 (10.9–19.0)	13.4 (9.9–18.0)	15.0 (11.1–19.3)	** < 0.001**
Fibrinogen (mg/dL)	215 (164–277)	212 (157–271)	216 (166–279)	**0.048**
D–dimer (μg/mL)	14.3 (5.7–31.4)	15.4 (5.1–27.5)	14.1 (5.9–33.4)	0.456
Lactate (mmol/L)	13.0 (10.2–16.0)	13.5 (10.6–16.4)	12.8 (10.1–15.9)	**0.031**
Rhythm before ECMO initiation				** < 0.001**
Shockable	853 (52.7)	202 (56.3)	651 (51.7)	
PEA	520 (32.1)	128 (35.7)	392 (31.1)	
Asystole	246 (15.2)	29 (8.1)	217 (17.2)	
ECMO cannulation place				** < 0.001**
Emergency room	1038 (64.0)	182 (50.7)	856 (67.8)	
Catheter laboratory	580 (35.8)	176 (49.0)	404 (32.0)	
Others	3 (0.2)	1 (0.3)	2 (0.2)	
Blood transfusion^e^				
Red blood cells, rate	832(64.3)	283(84.2)	549(57.3)	** < 0.001**
Red blood cells, Unit	4 (0–10)	10 (4–18)	2 (0–8)	** < 0.001**
Fresh frozen plasma, Unit	4 (0–10)	10 (3.3–16)	0 (0–8)	** < 0.001**
Platelet concentrations, Unit	0 (0–0)	0 (0–20)	0 (0–0)	** < 0.001**
Coronary angiography	1281 (78.5)	322 (89.4)	959 (75.5)	** < 0.001**
Percutaneous catheter intervention	754 (46.5)	207 (57.5)	547 (43.4)	** < 0.001**
Hemostasis procedure	79 (4.8)	79 (21.8)	–	
Interventional radiology (IVR)	19 (1.2)	19 (5.3)	–	
Surgical intervention	54 (3.3)	54 (15.0)	–	
IVR and surgical intervention	6 (0.4)	6 (1.7)	–	
Etiology of cardiac arrest				** < 0.001**
Cardiac causes of arrest	1410 (86.4)	323 (89.5)	1,087 (85.5)	
Acute coronary syndrome	967 (59.3)	240 (66.5)	727 (57.2)	
Arrhythmia	229 (14.0)	39 (10.8)	190 (15.0)	
Myopathy	96 (5.9)	23 (6.4)	73 (5.8)	
Myocarditis	19 (1.2)	5 (1.4)	14 (1.1)	
Other cardiac causes	99 (6.1)	16 (4.4)	83 (6.5)	
Non-cardiac causes arrest	106 (6.5)	27 (7.5)	79 (6.2)	
Pulmonary embolism	59 (3.6)	17 (4.7)	42 (3.3)	
Other non-cardiac causes	47 (2.9)	10 (2.9)	37 (2.9)	
Unknown	115 (7.1)	11 (3.1)	104 (8.2)	
Length of ICU stay, days	3 (1–10)	7 (2–13)	2 (1–9)	** < 0.001**
Length of hospital stay, days	4 (1–21)	9 (2–29)	3 (1–16)	** < 0.001**
Favorable neurological outcome at hospital discharge	230 (14.1)	50 (13.9)	180 (14.2)	0.881
Survival at hospital discharge	447 (27.4)	126 (34.9)	321 (25.3)	** < 0.001**

^a^Data are presented as median (interquartile range) for continuous variables and n (%) for categorical variables

^b^Bleeding complications were defined as conditions requiring blood transfusion or surgical intervention/interventional radiology (IVR), and cerebral hemorrhage was confirmed on computed tomography

^c^Call ambulance to arrival time is the time from call to emergency medical services to hospital arrival

^d^Estimated low flow time is the time from cardiac arrest to the establishment of ECMO if the location was in an ambulance and the time from calling an ambulance to the establishment of ECMO if cardiac arrest happened in other location

^e^Blood transfusion volume was calculated from the time of hospital arrival until 48 h after ICU admission

CPR, cardiopulmonary resuscitation; ECMO, extracorporeal membrane oxygenation; ICU, intensive care unit

Missing date: age = 1, Comorbidity = 80, Medication = 221, Location of cardiac arrest = 5, Witnessed cardiac arrest = 9, Bystander CPR = 25, Initial cardiac rhythm = 16, Time from ambulance to arrival = 26, Estimated low flow time = 79, Hemoglobin = 45, Platelet = 47, Fibrinogen = 201, D-dimer = 912, Lactate = 169, Rhythm before ECMO = 16, ECMO cannulation place = 11, Red blood cell = 338, Fresh frozen plasma = 353, Platelet concentrations = 401, Coronary angiography = 1, Percutaneous coronary intervention = 12, Etiology of cardiac arrest = 1, Length of ICU stay = 12, Length of hospital stay = 8

### Incidence rate of bleeding complications during hospitalization

**Fig. 2:**
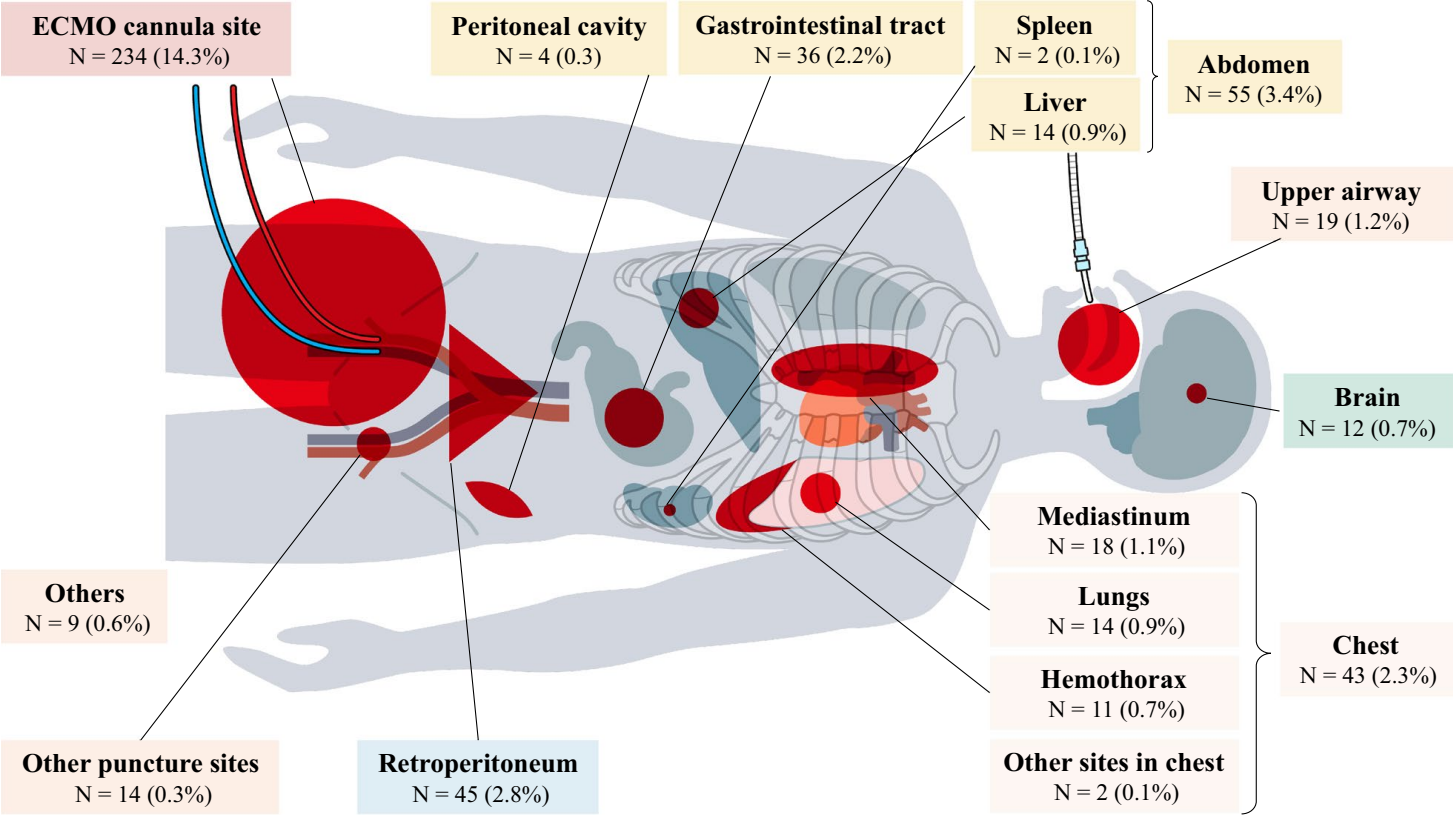
Bleeding complications in each organ. N = numbers

The total incidence of bleeding complications during hospital stay was 22.1% (431/361 patients). The most common bleeding sites were ECMO cannulation sites (14.3%), followed by the retroperitoneum (2.8%), gastrointestinal tract (2.2%), upper airway (1.2%), and mediastinum (1.1%) (Fig. 2).

### Timing of onset bleeding complications during hospitalization

**Fig. 3:**
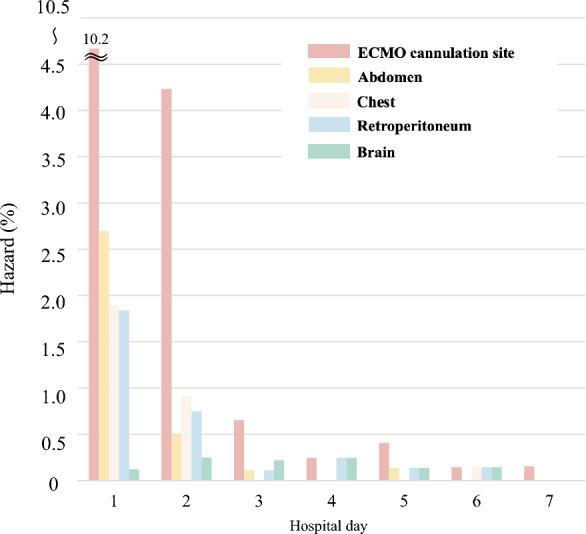
Hazard of bleeding complications. ECMO: extracorporeal membrane oxygenation. The hazards of bleeding complications at major bleeding sites were calculated as proportions of patients with bleeding complications on the day among those who were alive at the beginning of the day

Changes in seven-day hazards of bleeding complications for each bleeding site are shown in Fig. 3. The highest incidence of bleeding occurred on the first day, except for cerebral hemorrhage. After the fourth day, the hazard for each bleeding site progressively decreased to < 0.5%, but only ECMO cannulation site bleeding slightly increased on the fifth day.

### Risk factors for bleeding complications during the first day of admission

In a univariate analysis of the bleeding and non-bleeding groups during the first day of admission, significant differences were observed in terms of platelet counts and ECMO cannulation place, as shown in Additional file 1: Table S1. The mixed-effects logistic regression analysis showed a significant difference in terms of platelet count (< 10 × 10^4^/μL; adjusted OR: 1.865 [1.252–2.777], p = 0.002). (Table 2). By using composite outcome including bleeding complication and death, initial platelet count remained significant difference, and the result of the competing risk analysis accounting for early death was also determined the significant difference of initial platelet count (Sub-hazard ratio: 1.72[1.33ー2.22], p < 0.001). These results are shown in Additional file 1: Table S2, S3 and Figure S1.

**Table 2:** Multivariable analysis of factors associated with bleeding complications within the first day of admission among out-of-hospital cardiac arrest patients who received ECPR

	Odds ratio	95% confidence interval	P-value
Age (≧65 y/o)	1.221	0.868ー1.717	0.252
Hypertension	0.778	0.534ー1.135	0.192
Diabetes mellitus	0.990	0.655ー1.498	0.963
Dyslipidemia	1.439	0.878ー2.357	0.149
Cardiovascular disease	0.851	0.555ー1.305	0.460
Cerebrovascular disease	1.492	0.834ー2.670	0.178
Chronic renal failure	0.796	0.370ー1.713	0.149
Prior antiplatelet or/and anticoagulant	1.015	0.671ー1.536	0.943
Estimated low flow time (> 60 min)	0.869	0.608ー1.536	0.440
Hemoglobin (< 10 g/dL)	0.980	0.621ー1.545	0.930
Platelet (< 10 × 10^4^/μL)	1.865	1.252ー2.777	**0.002**
Fibrinogen (< 200 mg/dL)	0.831	0.579ー1.194	0.317
ECMO cannulation place (Other non-emergency room)	1.217	0.732ー2.024	0.450

ECMO, extracorporeal membrane oxygenation

### Transfusions and interventions required for bleeding complications within the first 48 h of admission

About 64.3% received red blood cell transfusions from hospital arrival until 48 h after ICU admission (Table 1). The median transfusion volumes of red blood cells, fresh frozen plasma, and platelets were 4 units (IQR, 0–10), 4 units (IQR, 0–10), and 0 units (IQR, 0–0), respectively (Table 1). The median total blood transfusion volume per bleeding site was the highest for liver bleeding at 73 units (IQR, 29.5–136), followed by intraperitoneal bleeding at 41 units (IQR, 14–54) and hemothorax at 40 units (IQR, 22–96). For hemostasis, IVR was required in 25 patients (1.2%), 60 patients (3.3%) required surgical interventions, and 6 patients (0.4%) required both. Among 361 patients with bleeding complications during their hospital stay, 21.9% required IVR or/and surgical interventions for hemostasis. Among the 25 patients who required IVR, 8 (32.0%) had mediastinal bleeding, 7 (28.0%) had liver bleeding, 3 (12.0%) had retroperitoneal bleeding, and 3 (12.0%) had hemothorax. Of the 60 patients who required surgical hemostasis, cannulation site bleeding occurred most frequently (41 patients, 68.3%), followed by liver bleeding in 6 patients (10.0%) and mediastinal bleeding in 4 patients (6.7%) (Table 3).

**Table 3:** Blood transfusions and interventions required for bleeding complications within the first 48 h because information on the exact volume of blood transfused was only obtained during this time in this registry

	RBCs ^a^ transfusion during the first 48 h(U)	FFP ^a^ transfusion during the first 48 h (U)	PCs ^b^ transfusion during the first 48 h (U)	Total ^c^ transfusion during the first 48 h (U)	IVR n = 25	Surgical intervention n = 60
Canula site bleeding (n = 234)	10 (4–18)	8 (4–16)	0 (0–10)	20 (9.5–44.5)	2 (8.0)	41 (68.3)
Retroperitoneal bleeding (n = 45)	12 (6–24)	15 (7.5–22.5)	0 (0–20)	34 (20–68)	3 (12.0)	3 (5.0)
Puncture site bleeding except ECMO cannulation site (n = 14)	8 (3.5–14)	9 (4–12)	0 (0–17.5)	24 (8.5–35.75)	2 (8.0)	2 (3.3)
Cerebral hemorrhage (n = 12)	12 (0–18)	11 (0–16.5)	0 (0–22.5)	30 (0–46)	0(0)	1 (1.9)
Upper airway bleeding (n = 19)	9 (6–14)	9.5 (1.5–14.5)	0 (0–5)	21 (8–30.5)	0(0)	1 (1.9)
Chest (n = 43)					12 (48.0)	6 (13.0)
Mediastinal bleeding (n = 18)	12 (4.5–24)	20 (10–28)	0 (0–50)	28 (14.5–102.5)	8 (32.0)	4 (6.7)
Hemothorax (n = 11)	18 (12–30)	10 (4–32)	20 (0–20)	40 (22–96)	3 (12.0)	2 (3.7)
Pulmonary hemorrhage (n = 14)	8 (5–14)	6 (3–17)	0 (0–0)	14 (9–28)	1 (4.0)	0 (0)
Others (n = 2)	15 (12–18)	17 (16–18)	0 (0–0)	32 (30–34)	0(0)	1 (1.9)
Abdomen (n = 55)					8 (32.0)	6 (11.1)
Gastrointestinal tract bleeding (n = 36)	8 (4–15.5)	10 (4–16)	0 (0–18.8)	22 (4.5–40)	0(0)	0 (0)
Liver bleeding (n = 14)	26 (14.5–38)	24 (11.5–52)	20 (0–35)	73 (29.5–136)	7 (28.0)	6 (10.0)
Splenic bleeding (n = 2)	2 (0–4)	9 (6–12)	0 (0–0)	11 (6–16)	0(0)	0 (0)
Intraperitoneal bleeding (n = 4)	12 (10–14)	12 (4–12)	20 (0–30)	46 (14–54)	2 (8.0)	0 (0)
Others (n = 9)	6 (4–14)	10 (0–13)	0(0–15)	16 (5–47)	1 (4.0)	3 (5.6)

^a^One unit of blood product is derived from 200 mL of whole blood. One unit of RBC and FFP are 140 and 120 mL, respectively

^b^Ten units of blood product is derived from 400 mL of whole blood. Ten units of PCs is approximately 200 mL

^c^Total means the sum of RBCs, FFP, and PC units

Missing data: blood transfusion volume = 21

ECMO, extracorporeal membrane oxygenation; RBCs, red blood cells; FFP, fresh frozen plasma; PCs, platelet concentrates; IVR, interventional radiology

## Discussion

This study described the incidence rate, timing of onset and treatment of each bleeding site, and risk factors associated with bleeding complications occurring within the first day of admission in OHCA patients who received ECPR.

Bleeding is the most common complication among ECPR patients [22]. However, due to the limited sample size and single-center nature of many studies, the reported incidence rates varied widely (8–70%) [1, 2, 4–10, 13, 23–25]. Its incidence rate among 1,632 patients (median age 60, male 84.7%, antiplatelet or/and anticoagulant medication 15.8%, cardiac etiology 86.4%, PCI 46.5%, survival to hospital discharge 27.4%) with OHCA who received ECPR was 22.1%, and ECMO cannula site bleeding had the highest incidence (14.3%), followed by retroperitoneal bleeding (2.8%). In general, ECPR is associated with a higher risk of bleeding compared with cardiac ECMO due to CPR-related injuries and technical difficulties in puncturing due to the absence of a palpable pulse [22]. Aubron et al. reported bleeding events in 80% of patients with VA-ECMO, which are based on the bleeding definition of the Extracorporeal Life Support Organization (ELSO). The value is significantly higher than that of our findings. However, it is important to consider that the shorter duration of ECMO support in patients undergoing ECPR could reduce the recorded incidence of bleeding caused by early mortality before the bleeding complications are detected. Haas et al. reported a higher incidence rate of bleeding complications (31.3%) in 217 adult OHCA patients (median age 52, male 72.8%, percutaneous cannulation 71.0%, PCI 26.3%, survival hospital discharge 27.6%) who received ECPR using the ELSO registry data; ECMO cannulation site bleeding had the highest incidence (18.4%), followed by gastrointestinal bleeding (9.7%) compared with our study population. This variation can be affected by differences in patient characteristics and differences in the classification and definition of bleeding complications [26]. Haas et al. only assessed bleeding at sites recorded on the ELSO registry form, whereas the SAVE-J II registry examined all bleeding sites requiring blood transfusion; hence, our study provided a more detailed incidence rate of bleeding complications in ECPR patients using large real-world data. The classification and definition of bleeding complications should be standardized in future research and database registries to facilitate comparisons between different institutions and studies [12].

No studies reported the timing of onset of bleeding complications in OHCA patients who received ECPR. This study is the first to provide the daily hazards for each bleeding site. Except for cerebral bleeding, most bleeding from ECMO cannula site, the abdomen, chest, and retroperitoneum occurred within the first two days of ECMO initiation. CPR-related injuries were more apparent in ECPR patients than in conventional CPR patients possibly due to the extended survival with ECMO [27, 28], leading to increased hazards of bleeding within the first two days. Although the onset of bleeding from each site remained low (< 1.0%) beyond the third day, it was unclear whether these bleeding complications were exacerbations of an initial minor bleeding or new onset of major bleeding. Aubron et al. reported that the median onset of bleeding was on day 4, including those not limited to ECPR, with pre-bleeding APTT levels being associated with bleeding complications [29]. The bleeding beyond the third day may be related to prolonged APTT levels, with coagulopathy, and/or anticoagulation therapy after ECMO initiation. Our result provided additional knowledge for future studies.

In terms of the risk factors of bleeding during the first day of admission, initial platelet counts of < 10 × 10^4^ /μL compared with > 10 × 10^4^ /μL upon arrival were associated with an increased risk of bleeding. In addition, given the importance of early mortality in our study population, we conducted additional analyses by using a composite outcome that included both bleeding complications and death within the first day of hospitalization, and competing risk analysis accounting for early death within the first week of hospitalization. These results contribute to the robustness of the association between platelet count and bleeding complication. Only few studies examined the bleeding risk factors for ECPR, and in a retrospective analysis of Otani et al. on 102 OHCA patients who received ECPR, age, initial platelet count, and D-dimer levels were associated with major bleeding [10]. Hence, a lower initial platelet count may be a useful predictor of bleeding complications requiring blood transfusion. In patients with trauma who presented with ongoing major bleeding, the platelet threshold is 5.0 − 10 × 10^4^/μL [30]. Thus, patients with ECPR may also require maintaining platelet counts above this value. Further investigations regarding the target populations, timing, and platelet counts during platelet transfusion should be performed.

Several studies reported that 40–80% of patients who undergo ECPR require blood transfusions [2, 28, 35, 36], which is comparable to the 64.3% observed in our present study. The most common bleeding sites requiring IVR were the mediastinum, followed by the liver, while the most common site requiring surgical intervention was ECMO cannula sites. Overall, 21.9% of bleeding patients required interventions. Although recent improvements in ECMO cannulation techniques using echo and X-ray fluoroscopy decreased the incidence of cannulation site bleeding [2, 9], it can still be life-threatening [37, 38]. If compression hemostasis is ineffective, a prompt surgical intervention is necessary for homeostasis. ELSO published an interim consensus statement on ECPR, which recommends that CT scan should be performed immediately in all ECPR cases [39]. IVR was also suggested for embolization of lacerations of the liver and spleen. Further studies are needed to examine optimal strategies and multidisciplinary treatment for patients with internal organ damage [40].

This retrospective observational study has several limitations. First, since this is a retrospective cohort study with chart review, it is possible that a greater number of patients had bleeding complications than what was actually recorded. Second, there were no standardized indications and subsequent management practices among participating institutions, which may have influenced the occurrence of bleeding complications. Third, due to the nature of the SAVE-J II registry data, information on the type of ECMO device, the ECMO cannulation method, use of anticoagulants and antiplatelet upon arrival, transfusions after the acute phase, other surgical interventions not aimed at hemostasis, or direct causes of death were not available. Fourth, since most patients had missing D-dimer values, the association with bleeding complications could not be analyzed. Fifth, as this study does not specify whether each bleeding event was caused by CPR, related procedures, or occurred spontaneously, specific measures to reduce bleeding complications could not be provided. Sixth, the rate of early mortality in this data set is high, which could be a competing risk for bleeding complication. Thus, the association between bleeding complications during hospitalization and clinical outcomes, such as survival and favorable neurological function, at hospital discharge was challenging to analyze.

## Conclusions

We described the details of bleeding complications associated with ECPR in OHCA patients using large registry data from 36 institutions in Japan. Approximately 20% of OHCA patients who received ECPR developed bleeding complications, of which 20% required IVR and/or surgical intervention for hemostasis. Almost all bleeding complications developed within two days of admission, while bleeding at ECMO cannulation sites remained low beyond the third day. A decreased platelet count (< 10 × 10^4^/μL compared with > 10 × 10^4^/μL) upon arrival was an independent risk factor of bleeding complications within the first day of admission. It is necessary to improve its safety to decrease the occurrence of bleeding complications.

### Supplementary Information

**Additional file 1.** Additional tables and figures.

## Data Availability

The datasets used and analyzed during the current study are available from the authors upon reasonable request.

## Abbreviations

CPR: Cardiopulmonary resuscitation
ECPR: Extracorporeal cardiopulmonary resuscitation
ELSO: Extracorporeal Life Support Organization
EMS: Emergency medical service
ICU: Intensive care unit
IVR: Interventional radiology
OHCA: Of-hospital cardiac arrest
OR: Odds ratio
PCI: Percutaneous catheter intervention
ROSC: Return of spontaneous circulation
