# Supplementary material for: Risk factors for bleeding complications in patients undergoing extracorporeal cardiopulmonary resuscitation following out-of-hospital cardiac arrest: a secondary analysis of the SAVE-J II study
Source: Ann Intensive Care. 2024 Jan 28;14:16. doi: 10.1186/s13613-024-01253-x (PMC10821854; doi:10.1186/s13613-024-01253-x)
Supplement: Supplementary file 1 — Additional file 1. Additional tables and figures. [file 13613_2024_1253_MOESM1_ESM.docx]

**Additional File: 1**

**Table S1**

**Characteristics of patients with and without bleeding complications within the first day of admission**

|  | **All n = 1,187** | **Bleeding n = 201** | **Non-Bleeding n = 986** | **P-value** |
| --- | --- | --- | --- | --- |
| Age, years | 60 (49ー68) | 62 (50ー70) | 60 (49ー68) | 0.104 |
| ≧65 | 449 (37.8) | 87 (43.3) | 362 (36.7) | 0.095 |
| Hypertension | 364 (30.7) | 61 (30.3) | 303 (30.7) | 0.982 |
| Diabetes mellitus | 244 (20.6) | 44 (21.9) | 200 (20.3) | 0.676 |
| Dyslipidemia | 145 (12.2) | 31 (15.4) | 200 (20.3) | 0.160 |
| Cardiovascular disease | 314 (26.5) | 54 (26.9) | 260 (26.4) | 0.954 |
| Cerebrovascular disease | 80 (6.7) | 19 (9.5) | 61 (6.2) | 0.126 |
| Chronic renal failure | 56 (4.7) | 10 (5.0) | 46 (4.7) | 0.995 |
| Medication |  |  |  | 0.515 |
| No antiplatelet or anticoagulant | 969 (81.6) | 162 (80.6) | 807 (81.8) |  |
| Antiplatelet or anticoagulant | 190 (16.0) | 32 (15.9) | 158 (16.0) |  |
| Antiplatelet and anticoagulant | 28 (2.4) | 7 (3.5) | 21 (2.1) |  |
| Estimated low flow time (min) | 54 (45–64) | 53 (43ー64) | 54 (45–64) | 0.517 |
| >60 | 395 (33.3) | 64 (31.8) | 331 (33.6) | 0.695 |
| Hemoglobin (g/dL) | 12.8 (11.0ー14.5) | 12.9 (10.8ー14.5) | 12.8 (11.1ー14.5) | 0.659 |
| <10 | 184 (15.5) | 37 (18.4) | 147 (14.9) | 0.253 |
| Platelet (×10^4^/μL) | 14.8 (10.9ー19.0) | 12.9 (9.4ー17.4) | 15.0 (11.2ー19.2) | **0.001** |
| <10 | 234 (19.7) | 56 (27.9) | 178 (18.1) | **0.002** |
| Fibrinogen (mg/dL) | 213 (164ー274) | 213 (160ー258) | 213 (165ー275) | 0.154 |
| <200 | 480 (40.4) | 81 (40.3) | 399 (40.5) | 1.000 |
| ECMO cannulation place |  |  |  | **0.001** |
| Emergency room | 782 (65.9) | 112 (55.7) | 670 (68.0) |  |
| Other non-emergency room | 405 (34.1) | 89 (44.3) | 316 (32.0) |  |

All data are analyzed in complete cases, so missing values are excluded.

**Table S2**

**Multivariable analysis of factors associated with composite outcome including bleeding complication and death within the first day among out-of-hospital cardiac arrest patients who received ECPR**

|  | **Odds ratio** | **95% confidence interval** | **P-value** |
| --- | --- | --- | --- |
| Age (≧65 y/o) | 1.630 | 1.243ー2.137 | **<0.001** |
| Hypertension | 0.832 | 0.615ー1.124 | 0.230 |
| Diabetes mellitus | 0.741 | 0.526ー1.043 | 0.086 |
| Dyslipidemia | 1.070 | 0.703ー1.629 | 0.753 |
| Cardiovascular disease | 1.280 | 0.914ー1.794 | 0.151 |
| Cerebrovascular disease | 1.439 | 0.870ー2.380 | 0.156 |
| Chronic renal failure | 0.929 | 0.499ー1.729 | 0.816 |
| Prior antiplatelet or/and anticoagulant | 0.797 | 0.568ー1.119 | 0.190 |
| Estimated low flow time (> 60 min) | 1.433 | 1.087ー1.889 | **0.011** |
| Hemoglobin (<10 g/dL) | 1.114 | 0.769ー1.613 | 0.568 |
| Platelet (<10×10*4/μL) | 1.826 | 1.316ー2.533 | **<0.001** |
| Fibrinogen (<200 mg/dL) | 1.161 | 0.876ー1.539 | 0.298 |
| ECMO cannulation place (Other non-emergency room) | 0.807 | 0.538ー1.211 | 0.301 |

ECMO, extracorporeal membrane oxygenation

**Table S3**

**Competitive risk analysis of factors associated with bleeding complication within 7 days among out-of-hospital cardiac arrest patients who received ECPR**

|  | **Sub-hazard**  **Ratio** | **95% confidence interval** | **P-value** |
| --- | --- | --- | --- |
| Age (≧65 y/o) | 1.10 | 0.87ー1.38 | 0.43 |
| Hypertension | 0.93 | 0.72ー1.20 | 0.59 |
| Diabetes mellitus | 0.99 | 0.74ー1.31 | 0.94 |
| Dyslipidemia | 1.27 | 0.79ー1.39 | 0.15 |
| Cardiovascular disease | 1.04 | 0.78ー1.77 | 0.79 |
| Cerebrovascular disease | 1.50 | 0.67ー1.031 | **0.03** |
| Chronic renal failure | 0.63 | 0.35ー1.15 | 0.13 |
| Prior antiplatelet or/and anticoagulant | 0.92 | 0.68ー1.24 | 0.58 |
| Estimated low flow time (> 60 min) | 0.98 | 0.78ー1.23 | 0.85 |
| Hemoglobin (<10 g/dL) | 0.91 | 0.68ー1.23 | 0.54 |
| Platelet (<10×10*4/μL) | 1.72 | 1.33ー2.22 | **<0.001** |
| Fibrinogen (<200 mg/dL) | 0.87 | 0.68ー1.11 | 0.26 |
| ECMO cannulation place (Other non-emergency room) | 1.68 | 1.35ー2.09 | **<0.001** |

ECMO, extracorporeal membrane oxygenation

**Figure S1 Cumulative bleeding complication rate for a week for the two groups divided by initial platelet count of 100,000/μL**
